# Supplementary material for: Deletion of wfs1 Impairs Oligodendrocyte Precursor Cells Dorsal Distribution and Myelination Through the wfs1-hmgcs1 Axis in Zebrafish
Source: Biology (Basel). 2026 Aug 16;15(16):1402. doi: 10.3390/biology15161402 (PMC13510151; doi:10.3390/biology15161402)
Supplement: Supplementary file 1 [file biology-15-01402-s001.zip › File S1-Transcriptome sequencing data and differentially expressed genes (DEGs) in wfs1-- zebrafish at 5 dpf/2.KEGG/wfs1KOvsWT/UP/wfs1KOvsWT.up_KEGGenrich.html]

Pathway Enrichment


# The most enriched pathway terms

Statistic method: hypergeometric test

FDR correction method: Benjamini and Hochberg

| Term | Description | Sample number | Background number | P-value | Corrected P-value | Gene\_id | Gene\_name | KEGG\_ID |
| --- | --- | --- | --- | --- | --- | --- | --- | --- |
| dre04672 | Intestinal immune network for IgA production | 8/511 | 37/8379 | 0.00144979704036775 | 0.221818947176266 | 799354 30762 368878 30763 571282 100006078 100151365\_1 100333914 | cxcl12b mhc2dab si:busm1-266f07.2 mhc2dcb LOC571282 ccr9b si:dkey-1h24.6 ccl25a | dre:799354 dre:30762 dre:368878 dre:30763 dre:571282 dre:100006078 dre:100151365 dre:100333914 |
| dre00270 | Cysteine and methionine metabolism | 11/511 | 73/8379 | 0.00442061152121748 | 0.321646522266322 | 335715 334195\_1 103911233 317744\_1 561410\_1 445818 431773\_1 30659\_1 103911241 563642 450084\_1 | mdh1ab mat1a LOC103911233 dnmt3bb.1 tat cthl enoph1 dnmt3bb.2 LOC103911241 sdsl gss | dre:335715 dre:334195 dre:103911233 dre:317744 dre:561410 dre:445818 dre:431773 dre:30659 dre:571201 dre:563642 dre:450084 |
| dre04130 | SNARE interactions in vesicular transport | 9/511 | 56/8379 | 0.00630679455424161 | 0.321646522266322 | 567385\_1 449923 436605 449923\_1 393836 571872 325484 393236 565822\_1 | vti1a si:ch73-234b20.5 stx5a si:ch73-234b20.5 stx11b.1 zgc:165520 vamp5 sybl1 bnip1a | dre:567385 dre:449923 dre:436605 dre:449923 dre:393836 dre:571872 dre:325484 dre:393236 dre:565822 |
| dre01200 | Carbon metabolism | 19/511 | 173/8379 | 0.00898588128128341 | 0.34370995900909 | 335715 560753\_1 116517 445233 560827\_1 406762\_1 403070\_1 437019\_1 541329 570579\_1 100006589\_1 550579\_1 561416\_1 317746 406702 563642 321224 751668 568001\_3 | mdh1ab tpi1b adh5 me2 prps1b pgd rgn pcca gcsha g6pd idh1 idh3g pfkpb suclg2 pdha1a sdsl hkdc1 gck pfkmb | dre:335715 dre:560753 dre:116517 dre:445233 dre:560827 dre:406762 dre:403070 dre:437019 dre:541329 dre:570579 dre:100006589 dre:550579 dre:561416 dre:317746 dre:406702 dre:563642 dre:321224 dre:751668 dre:568001 |
| dre03018 | RNA degradation | 13/511 | 108/8379 | 0.0139295072647707 | 0.426242922301983 | 373085\_1 448949\_1 394064\_1 553665\_1 100538016 492479\_1 565000 561416\_1 317641\_1 566017\_1 565659\_1 321035\_1 568001\_3 | hspa9 cnot1 exosc10 cnot6l si:dkey-79d12.5 btg3 exosc3 pfkpb zcchc7 lsm8 edc4 pabpc4 pfkmb | dre:373085 dre:448949 dre:394064 dre:553665 dre:100538016 dre:492479 dre:324574 dre:561416 dre:317641 dre:566017 dre:565659 dre:321035 dre:568001 |
| dre03440 | Homologous recombination | 7/511 | 46/8379 | 0.0203143738784852 | 0.449686643356765 | 558923 406487 404608 794038 406721 554171\_1 450081 | palb2 rad51 rad51d brip1 rad51b rad52 rad51c | dre:558923 dre:406487 dre:404608 dre:794038 dre:406721 dre:554171 dre:450081 |
| dre00590 | Arachidonic acid metabolism | 9/511 | 72/8379 | 0.0300955357920534 | 0.449686643356765 | 406449\_1 494108 559020 393297 406370 566996 406575\_1 393592 560649 | ptges3a ptgr1 ptgs2b zgc:56585 pla2g6 si:dkey-11k2.7 lta4h cyp2p9 si:ch211-214p16.3 | dre:406449 dre:494108 dre:559020 dre:393297 dre:406370 dre:566996 dre:406575 dre:393592 dre:560649 |
| dre00470 | D-Amino acid metabolism | 3/511 | 12/8379 | 0.0328172489607309 | 0.449686643356765 | 560387 405800 619259 | c17h14orf159 dao.2 dao.1 | dre:560387 dre:405800 dre:619259 |
| dre02010 | ABC transporters | 8/511 | 63/8379 | 0.0365537783902772 | 0.449686643356765 | 368620\_1 570453\_1 406803\_1 406803\_2 571189 570148\_1 735310 556826\_1 | abcc4 abcg2b abcd3a abcd3a abcb11b abcb9 abcg2d si:dkey-57h18.2 | dre:368620 dre:570453 dre:406803 dre:406803 dre:571189 dre:570148 dre:735310 dre:556826 |
| dre00360 | Phenylalanine metabolism | 4/511 | 22/8379 | 0.0416424711150968 | 0.449686643356765 | 541429 561410\_1 445287 394142 | aoc2 tat hpdb hpda | dre:541429 dre:561410 dre:445287 dre:394142 |
| dre00450 | Selenocompound metabolism | 4/511 | 22/8379 | 0.0416424711150968 | 0.449686643356765 | 103911233 101884223 445818 103911241 | LOC103911233 LOC101884223 cthl LOC103911241 | dre:103911233 dre:101884223 dre:445818 dre:571201 |
| dre00500 | Starch and sucrose metabolism | 6/511 | 44/8379 | 0.0491876345987772 | 0.449686643356765 | 393444 406539 445049 100333665 321224 751668 | pygmb amy2a zgc:92137 gbe1a hkdc1 gck | dre:393444 dre:406539 dre:445049 dre:100333665 dre:321224 dre:751668 |
| dre00130 | Ubiquinone and other terpenoid-quinone biosynthesis | 3/511 | 14/8379 | 0.049615023389686 | 0.449686643356765 | 561410\_1 445287 394142 | tat hpdb hpda | dre:561410 dre:445287 dre:394142 |
| dre00260 | Glycine, serine and threonine metabolism | 7/511 | 56/8379 | 0.0524806097147263 | 0.449686643356765 | 541429 541329 445818 394103 405800 619259 563642 | aoc2 gcsha cthl sardh dao.2 dao.1 sdsl | dre:541429 dre:541329 dre:445818 dre:394103 dre:405800 dre:619259 dre:563642 |
| dre00620 | Pyruvate metabolism | 8/511 | 68/8379 | 0.0537296369566022 | 0.449686643356765 | 335715 116517 445233 406473 368239 559403\_1 406702 445326\_1 | mdh1ab adh5 me2 pck2 aldh2.2 acaca pdha1a akr1a1a | dre:335715 dre:116517 dre:445233 dre:406473 dre:368239 dre:559403 dre:406702 dre:445326 |
| dre00030 | Pentose phosphate pathway | 6/511 | 45/8379 | 0.0539635980559191 | 0.449686643356765 | 560827\_1 406762\_1 403070\_1 570579\_1 561416\_1 568001\_3 | prps1b pgd rgn g6pd pfkpb pfkmb | dre:560827 dre:406762 dre:403070 dre:570579 dre:561416 dre:568001 |
| dre00100 | Steroid biosynthesis | 4/511 | 24/8379 | 0.0550048729176227 | 0.449686643356765 | 322481 100004700 565117 406662\_2 | cel.1 cyp24a1 cel.2 msmo1 | dre:322481 dre:100004700 dre:565117 dre:406662 |
| dre04070 | Phosphatidylinositol signaling system | 15/511 | 157/8379 | 0.0554687780397886 | 0.449686643356765 | 100002015\_1 336121 724010\_1 368717 337236\_2 559938\_1 100000252\_3 100006675 337236 566642\_1 571356 678558\_1 571356\_2 100000252 559198 | cds1 calm2a dgkaa itpka synj1 ppip5k1b pik3r3b prkcba synj1 tmem55a pik3c2a mtmr7a pik3c2a pik3r3b mtmr7b | dre:100002015 dre:192322 dre:724010 dre:368717 dre:337236 dre:559938 dre:100000252 dre:100006675 dre:337236 dre:566642 dre:571356 dre:678558 dre:571356 dre:100000252 dre:559198 |
| dre01232 | Nucleotide metabolism | 13/511 | 131/8379 | 0.0558434393711015 | 0.449686643356765 | 100330864 334431\_1 492517 550560\_1 100005763 103910413\_1 393744\_1 553639\_1 402953\_1 333997 558466\_1 447905 333997\_1 | LOC100330864 adsl zgc:103586 entpd6 uck2a tymp dctpp1 gmpr pnp6 ampd3b uckl1b entpd2a.1 ampd3b | dre:100330864 dre:334431 dre:492517 dre:550560 dre:100005763 dre:103910413 dre:393744 dre:553639 dre:402953 dre:333997 dre:558466 dre:447905 dre:333997 |
| dre00020 | Citrate cycle (TCA cycle) | 6/511 | 47/8379 | 0.0643307133264553 | 0.492129956947383 | 335715 406473 100006589\_1 550579\_1 317746 406702 | mdh1ab pck2 idh1 idh3g suclg2 pdha1a | dre:335715 dre:406473 dre:100006589 dre:550579 dre:317746 dre:406702 |
| dre00900 | Terpenoid backbone biosynthesis | 4/511 | 26/8379 | 0.0704047356283084 | 0.512948788149104 | 334380\_1 559054\_1 557720\_1 100003383 | zmpste24 hmgcra rce1a pmvk | dre:334380 dre:559054 dre:557720 dre:100003383 |
| dre00350 | Tyrosine metabolism | 6/511 | 50/8379 | 0.0819215629038307 | 0.569727232922095 | 116517 541429 561410\_1 445287 394142 30505 | adh5 aoc2 tat hpdb hpda dbh | dre:116517 dre:541429 dre:561410 dre:445287 dre:394142 dre:30505 |
| dre03460 | Fanconi anemia pathway | 7/511 | 63/8379 | 0.087549069400731 | 0.582391635578776 | 100538153 558923 406487 794038 402936\_1 450081 570043\_1 | slx4 palb2 rad51 brip1 fancg rad51c rev1 | dre:100538153 dre:558923 dre:406487 dre:794038 dre:402936 dre:450081 dre:570043 |
| dre00010 | Glycolysis / Gluconeogenesis | 10/511 | 103/8379 | 0.0966016735599436 | 0.615835668944641 | 560753\_1 116517 406473 368239 561416\_1 406702 445326\_1 321224 751668 568001\_3 | tpi1b adh5 pck2 aldh2.2 pfkpb pdha1a akr1a1a hkdc1 gck pfkmb | dre:560753 dre:116517 dre:406473 dre:368239 dre:561416 dre:406702 dre:445326 dre:321224 dre:751668 dre:568001 |
| dre03430 | Mismatch repair | 4/511 | 30/8379 | 0.106986131418301 | 0.65037961238244 | 556995\_1 565400 568323\_1 565400\_1 | lig1 msh3 mlh3 msh3 | dre:556995 dre:565400 dre:568323 dre:565400 |
| dre00380 | Tryptophan metabolism | 7/511 | 67/8379 | 0.112362499342447 | 0.65037961238244 | 103911233 447801\_1 368239 393860 571967 555401 103911241 | LOC103911233 aldh8a1 aldh2.2 gcdha ido1 aoc1 LOC103911241 | dre:103911233 dre:447801 dre:368239 dre:393860 dre:571967 dre:555401 dre:571201 |
| dre00052 | Galactose metabolism | 5/511 | 43/8379 | 0.118918974932763 | 0.65037961238244 | 561416\_1 450083 321224 751668 568001\_3 | pfkpb gla hkdc1 gck pfkmb | dre:561416 dre:450083 dre:321224 dre:751668 dre:568001 |
| dre00603 | Glycosphingolipid biosynthesis - globo and isoglobo series | 3/511 | 20/8379 | 0.119023719913126 | 0.65037961238244 | 447881 450083 100005375 | gbgt1l3 gla si:ch211-135f11.4 | dre:447881 dre:450083 dre:100005375 |
| dre01230 | Biosynthesis of amino acids | 10/511 | 109/8379 | 0.12742603967 | 0.672282209293449 | 334195\_1 560753\_1 560827\_1 100006589\_1 550579\_1 393799 561416\_1 445818 563642 568001\_3 | mat1a tpi1b prps1b idh1 idh3g pycr1a pfkpb cthl sdsl pfkmb | dre:334195 dre:560753 dre:560827 dre:100006589 dre:550579 dre:393799 dre:561416 dre:445818 dre:563642 dre:568001 |
| dre00591 | Linoleic acid metabolism | 4/511 | 33/8379 | 0.138978387655627 | 0.708789777043695 | 406370 393592 553969 560649 | pla2g6 cyp2p9 cyp3a65 si:ch211-214p16.3 | dre:406370 dre:393592 dre:553969 dre:560649 |
| dre00480 | Glutathione metabolism | 8/511 | 85/8379 | 0.145697001483808 | 0.719085200871697 | 100330864 406762\_1 570579\_1 100006589\_1 555317 393493 431762\_1 450084\_1 | LOC100330864 pgd g6pd idh1 si:ch211-276a23.5 gstt2 mgst1.2 gss | dre:100330864 dre:406762 dre:570579 dre:100006589 dre:555317 dre:393493 dre:431762 dre:450084 |
| dre00561 | Glycerolipid metabolism | 8/511 | 89/8379 | 0.174522555745416 | 0.834435969657769 | 724010\_1 322481 368239 799099\_1 565117 450083 445326\_1 572841 | dgkaa cel.1 aldh2.2 lpin1 cel.2 gla akr1a1a mboat1 | dre:724010 dre:322481 dre:368239 dre:799099 dre:565117 dre:450083 dre:445326 dre:572841 |
| dre03266 | Virion - Herpesvirus | 2/511 | 13/8379 | 0.18602357444256 | 0.86247293605187 | 791704 564266 | zgc:153759 itgb3a | dre:791704 dre:564266 |
| dre00790 | Folate biosynthesis | 4/511 | 38/8379 | 0.199321932778826 | 0.896948697504717 | 557552 791448\_1 100330410 574009 | zgc:153031 qdprb1 alp3 alpi.2 | dre:557552 dre:791448 dre:100330410 dre:574009 |
| dre00592 | alpha-Linolenic acid metabolism | 3/511 | 26/8379 | 0.209223861664205 | 0.914607166703526 | 406370 140615 560649 | pla2g6 fads2 si:ch211-214p16.3 | dre:406370 dre:140615 dre:560649 |
| dre00565 | Ether lipid metabolism | 6/511 | 67/8379 | 0.223107328592827 | 0.923514467691524 | 406370 492735 565743\_1 560225 560649 567170\_1 | pla2g6 si:ch211-212o1.2 pld2 cept1a si:ch211-214p16.3 pafah1b2 | dre:406370 dre:492735 dre:565743 dre:560225 dre:560649 dre:567170 |
| dre00330 | Arginine and proline metabolism | 7/511 | 81/8379 | 0.223333564082264 | 0.923514467691524 | 321892\_1 368239 393799 405800 555401 619259 327506\_1 | ckmt1 aldh2.2 pycr1a dao.2 aoc1 dao.1 ckmt2a | dre:321892 dre:368239 dre:393799 dre:405800 dre:555401 dre:619259 dre:327506 |
| dre00051 | Fructose and mannose metabolism | 5/511 | 54/8379 | 0.231536361947845 | 0.932238509947901 | 560753\_1 561416\_1 436909 321224 568001\_3 | tpi1b pfkpb khk hkdc1 pfkmb | dre:560753 dre:561416 dre:436909 dre:321224 dre:568001 |
| dre04210 | Apoptosis | 17/511 | 232/8379 | 0.249407845435453 | 0.960130628213667 | 57934\_1 431763 556410\_1 100000252\_3 394198 570238 567021 569298 101886621\_1 768248 561021 552927 58133 431777 791531 100000252 100135062\_2 | actb1 gadd45ab atf4b pik3r3b fosab fosaa ern2 ctsbb LOC101886621 fas si:dkey-103e21.5 casp6 ngfb tuba7l casp6l1 pik3r3b csf2rb | dre:57934 dre:431763 dre:556410 dre:100000252 dre:394198 dre:570238 dre:567021 dre:569298 dre:101886621 dre:768248 dre:561021 dre:552927 dre:58133 dre:431777 dre:791531 dre:100000252 dre:100135062 |
| dre00240 | Pyrimidine metabolism | 7/511 | 84/8379 | 0.251014543323835 | 0.960130628213667 | 100330864 492517 550560\_1 100005763 103910413\_1 393744\_1 558466\_1 | LOC100330864 zgc:103586 entpd6 uck2a tymp dctpp1 uckl1b | dre:100330864 dre:492517 dre:550560 dre:100005763 dre:103910413 dre:393744 dre:558466 |
| dre00512 | Mucin type O-glycan biosynthesis | 4/511 | 43/8379 | 0.265907406891521 | 0.963031711752537 | 324510\_1 557675\_1 555344 337131 | gcnt4a c1galt1a si:dkey-202e17.1 c1galt1b | dre:324510 dre:557675 dre:555344 dre:337131 |
| dre04150 | mTOR signaling pathway | 18/511 | 251/8379 | 0.270502831646342 | 0.963031711752537 | 324140\_1 796322 436718 79380 799099\_1 359840 100000252\_3 100006675 553214\_1 100330617\_1 555584\_1 100000252 359839 245702 100009657\_1 556087 58072\_1 573492\_1 | sgk1 slc3a2a rhocb eif4ea lpin1 atp6v1b2 pik3r3b prkcba eif4ba tsc1b prr5a pik3r3b atp6v1ba igf1rb rps6ka3b wnt2bb fzd8b rragca | dre:324140 dre:796322 dre:436718 dre:79380 dre:799099 dre:359840 dre:100000252 dre:100006675 dre:553214 dre:100330617 dre:555584 dre:100000252 dre:359839 dre:245702 dre:100009657 dre:556087 dre:58072 dre:573492 |
| dre04137 | Mitophagy - animal | 9/511 | 116/8379 | 0.275779603292001 | 0.963031711752537 | 550134\_1 novel.905 556410\_1 555713 326974\_1 564996 492676 561933\_2 561933\_1 | ubb - atf4b nbr1b gabarapa tfe3b tbc1d15 rhot1b rhot1b | dre:550134 dre:777766 dre:556410 dre:555713 dre:326974 dre:564996 dre:492676 dre:561933 dre:561933 |
| dre04914 | Progesterone-mediated oocyte maturation | 10/511 | 133/8379 | 0.292654935428156 | 0.963031711752537 | 323509\_1 562552 767751\_1 100000252\_3 568726 100000252 560807 245702 100009657\_1 280649\_1 | gnaia zgc:171775 ccnb3 pik3r3b adcy7 pik3r3b adcy6b igf1rb rps6ka3b plk1 | dre:323509 dre:562552 dre:767751 dre:100000252 dre:568726 dre:100000252 dre:560807 dre:245702 dre:100009657 dre:280649 |
| dre04068 | FoxO signaling pathway | 15/511 | 210/8379 | 0.299809553004783 | 0.963031711752537 | 324140\_1 406473 117508\_1 431763 566841 562552 117508 767751\_1 100000252\_3 326974\_1 565576 100000252 245702 280649\_1 559723 | sgk1 pck2 klf2a gadd45ab crebbpa zgc:171775 klf2a ccnb3 pik3r3b gabarapa plk2b pik3r3b igf1rb plk1 tgfb2l | dre:324140 dre:406473 dre:117508 dre:431763 dre:566841 dre:562552 dre:117508 dre:767751 dre:100000252 dre:326974 dre:565576 dre:100000252 dre:245702 dre:280649 dre:559723 |
| dre04370 | VEGF signaling pathway | 8/511 | 104/8379 | 0.30062018233563 | 0.963031711752537 | 559020 562552 793158\_1 100000252\_3 100006675 556226\_1 565241 100000252 | ptgs2b zgc:171775 cdc42l pik3r3b prkcba cdc42l2 ppp3ccb pik3r3b | dre:559020 dre:562552 dre:793158 dre:100000252 dre:100006675 dre:556226 dre:565241 dre:100000252 |
| dre00730 | Thiamine metabolism | 2/511 | 18/8379 | 0.301193320203708 | 0.963031711752537 | 100330410 574009 | alp3 alpi.2 | dre:100330410 dre:574009 |
| dre04060 | Cytokine-cytokine receptor interaction | 17/511 | 242/8379 | 0.307256614422064 | 0.963031711752537 | 799354 368326\_1 58151\_1 561050\_1 799279 368438\_1 562247 768248 100135755 58133 791704 100006078 100135062\_2 100001424 100333914 30125 559723 | cxcl12b il13ra2 cxcl14 ackr3b LOC799279 cntfr clcf1 fas il6r ngfb zgc:153759 ccr9b csf2rb ghra ccl25a gdf3 tgfb2l | dre:799354 dre:368326 dre:58151 dre:561050 dre:799279 dre:368438 dre:562247 dre:768248 dre:100135755 dre:58133 dre:791704 dre:100006078 dre:100135062 dre:100001424 dre:100333914 dre:30125 dre:559723 |
| dre03320 | PPAR signaling pathway | 8/511 | 106/8379 | 0.318937338361953 | 0.963031711752537 | 550134\_1 406473 novel.905 334529 30355\_1 553512 140615 550432 | ubb pck2 - aqp7 apoa1a slc27a1b fads2 slc27a4 | dre:550134 dre:406473 dre:777766 dre:334529 dre:30355 dre:553512 dre:140615 dre:550432 |
| dre04142 | Lysosome | 15/511 | 214/8379 | 0.325549173534017 | 0.963031711752537 | 565099 100003043\_1 569298 450083 368911 436725\_1 553423 566517 445244 559575 327237\_1 570148\_1 406511\_1 393657 567720 | zgc:193725 lamp3 ctsbb gla laptm4b acp5b sumf1 aga ap1m3 si:ch211-15d5.11 ap1s2 abcb9 sort1a zgc:66024 idua | dre:565099 dre:100003043 dre:569298 dre:450083 dre:368911 dre:436725 dre:553423 dre:566517 dre:445244 dre:559575 dre:327237 dre:570148 dre:406511 dre:393657 dre:567720 |
| dre00562 | Inositol phosphate metabolism | 9/511 | 123/8379 | 0.335378437042998 | 0.963031711752537 | 560753\_1 368717 337236\_2 337236 100006223 571356 678558\_1 571356\_2 559198 | tpi1b itpka synj1 synj1 si:dkey-266f7.9 pik3c2a mtmr7a pik3c2a mtmr7b | dre:560753 dre:368717 dre:337236 dre:337236 dre:100006223 dre:571356 dre:678558 dre:571356 dre:559198 |
| dre04810 | Regulation of actin cytoskeleton | 27/511 | 404/8379 | 0.337123070521647 | 0.963031711752537 | 57934\_1 799354 570868\_1 286739 436718 566764\_1 793158\_1 100000252\_3 100331581 100002872\_2 677750 556226\_1 796490\_2 494456 100005469\_1 570832 553493\_1 30429 58075 100000252 794365 103909601 564565 798694\_1 570832\_1 564266 101882911 | actb1 cxcl12b itga1 msna rhocb scin cdc42l pik3r3b si:dkey-44g17.6 cyfip2 myl2b cdc42l2 pdgfbb fgf23 fn1a c7b arhgef7a mylpfa fgd pik3r3b si:dkey-163m14.7 LOC103909601 arhgap35 LOC798694 c7b itgb3a LOC101882911 | dre:57934 dre:799354 dre:570868 dre:286739 dre:436718 dre:566764 dre:793158 dre:100000252 dre:100331581 dre:100002872 dre:677750 dre:556226 dre:796490 dre:494456 dre:100005469 dre:570832 dre:553493 dre:30429 dre:58075 dre:100000252 dre:794365 dre:103909601 dre:564565 dre:798694 dre:570832 dre:564266 dre:321070 |
| dre04330 | Notch signaling pathway | 7/511 | 93/8379 | 0.339039240588408 | 0.963031711752537 | 678649\_1 566841 393500\_1 557340\_1 30120\_1 497616 140422 | zgc:136929 crebbpa zgc:66440 atxn1a dlc numbl jag2b | dre:678649 dre:566841 dre:393500 dre:557340 dre:30120 dre:497616 dre:140422 |
| dre05132 | Salmonella infection | 27/511 | 406/8379 | 0.346797465516346 | 0.963031711752537 | 57934\_1 386590\_1 562529\_1 100004870\_1 562372\_1 558917\_1 562552 436718 563023\_1 793158\_1 394198 570238 798985\_1 100002872\_2 101882034 677750 566185 393653\_1 556226\_1 571356 561021 571356\_2 110437957 548603\_1 431777 30429 101882911 | actb1 hsp90b1 flna exoc2 snx18a snx9b zgc:171775 rhocb flnb cdc42l fosab fosaa vps16 cyfip2 LOC101882034 myl2b caspbl rhogd cdc42l2 pik3c2a si:dkey-103e21.5 pik3c2a LOC110437957 snx18b tuba7l mylpfa LOC101882911 | dre:57934 dre:386590 dre:562529 dre:100004870 dre:562372 dre:558917 dre:562552 dre:436718 dre:563023 dre:793158 dre:394198 dre:570238 dre:798985 dre:100002872 dre:406297 dre:677750 dre:566185 dre:393653 dre:556226 dre:571356 dre:561021 dre:571356 dre:373874 dre:548603 dre:431777 dre:30429 dre:321070 |
| dre04625 | C-type lectin receptor signaling pathway | 11/511 | 156/8379 | 0.353750614534213 | 0.963031711752537 | 336121 559020 562552 436718 100000252\_3 368241 566185 556277 561021 565241 100000252 | calm2a ptgs2b zgc:171775 rhocb pik3r3b egr2a caspbl cblb si:dkey-103e21.5 ppp3ccb pik3r3b | dre:192322 dre:559020 dre:562552 dre:436718 dre:100000252 dre:368241 dre:566185 dre:556277 dre:561021 dre:565241 dre:100000252 |
| dre01240 | Biosynthesis of cofactors | 16/511 | 234/8379 | 0.354100385531963 | 0.963031711752537 | 334195\_1 334431\_1 557552 403070\_1 368239 445287 394142 571967 445083 393580 406789 445326\_1 110438097 100330410 574009 450084\_1 | mat1a adsl zgc:153031 rgn aldh2.2 hpdb hpda ido1 dhrs3a bco1l pank1a akr1a1a LOC110438097 alp3 alpi.2 gss | dre:334195 dre:334431 dre:557552 dre:403070 dre:368239 dre:445287 dre:394142 dre:571967 dre:445083 dre:393580 dre:406789 dre:445326 dre:572070 dre:100330410 dre:574009 dre:450084 |
| dre03013 | Nucleocytoplasmic transport | 9/511 | 129/8379 | 0.388059531884852 | 0.963031711752537 | 393693\_1 100005893 393973 324342\_1 406860 321744\_1 100004503 407710 333980 | sap18 magoh rae1 xpo4 ipo9 nup88 eef1a1b xpo7 xpo6 | dre:393693 dre:100005893 dre:393973 dre:324342 dre:406860 dre:321744 dre:100004503 dre:407710 dre:333980 |
| dre04910 | Insulin signaling pathway | 14/511 | 212/8379 | 0.418664545156926 | 0.963031711752537 | 100001260\_1 393444 406473 336121 79380 100000252\_3 556277 100330617\_1 559403\_1 100000252 321224 751668 406459 569706\_1 | ppp1r3ab pygmb pck2 calm2a eif4ea pik3r3b cblb tsc1b acaca pik3r3b hkdc1 gck zgc:77112 phkb | dre:100001260 dre:393444 dre:406473 dre:192322 dre:79380 dre:100000252 dre:556277 dre:100330617 dre:559403 dre:100000252 dre:321224 dre:751668 dre:406459 dre:569706 |
| dre01040 | Biosynthesis of unsaturated fatty acids | 3/511 | 39/8379 | 0.427944133054092 | 0.963031711752537 | 140615 393441 554145 | fads2 hacd3 elovl8b | dre:140615 dre:393441 dre:554145 |
| dre00310 | Lysine degradation | 7/511 | 102/8379 | 0.430312734433237 | 0.963031711752537 | 368239 393860 556077\_1 100003615 572649 402830 556535 | aldh2.2 gcdha plod3 prdm2a bbox1 ehmt1b setd1a | dre:368239 dre:393860 dre:556077 dre:100003615 dre:572649 dre:402830 dre:556535 |
| dre03015 | mRNA surveillance pathway | 8/511 | 118/8379 | 0.431542747341909 | 0.963031711752537 | 386630\_1 324267\_1 393693\_1 100005893 100002680\_2 321035\_1 559392\_1 562282 | tardbpl hbs1l sap18 magoh msi2a pabpc4 ppp2r2cb ppp2r5b | dre:386630 dre:324267 dre:393693 dre:100005893 dre:100002680 dre:321035 dre:559392 dre:562282 |
| dre00910 | Nitrogen metabolism | 2/511 | 24/8379 | 0.435064978206286 | 0.963031711752537 | 569989\_1 767680 | ca5a zgc:153760 | dre:569989 dre:767680 |
| dre00280 | Valine, leucine and isoleucine degradation | 5/511 | 71/8379 | 0.437554104207435 | 0.963031711752537 | 368239 437019\_1 394130\_1 797311 777632 | aldh2.2 pcca acad8 LOC797311 mccc1 | dre:368239 dre:437019 dre:394130 dre:797311 dre:777632 |
| dre00564 | Glycerophospholipid metabolism | 9/511 | 135/8379 | 0.441142125322699 | 0.963031711752537 | 100002015\_1 724010\_1 406370 799099\_1 334017\_1 565743\_1 560225 560649 572841 | cds1 dgkaa pla2g6 lpin1 pla1a pld2 cept1a si:ch211-214p16.3 mboat1 | dre:100002015 dre:724010 dre:406370 dre:799099 dre:334017 dre:565743 dre:560225 dre:560649 dre:572841 |
| dre03060 | Protein export | 2/511 | 25/8379 | 0.456096170206961 | 0.963031711752537 | 436794 445299\_1 | sec11a immp2l | dre:436794 dre:445299 |
| dre00640 | Propanoate metabolism | 3/511 | 41/8379 | 0.460604499470477 | 0.963031711752537 | 437019\_1 317746 559403\_1 | pcca suclg2 acaca | dre:437019 dre:317746 dre:559403 |
| dre00400 | Phenylalanine, tyrosine and tryptophan biosynthesis | 1/511 | 10/8379 | 0.467192894557882 | 0.963031711752537 | 561410\_1 | tat | dre:561410 |
| dre05168 | Herpes simplex virus 1 infection | 14/511 | 220/8379 | 0.47463801701342 | 0.963031711752537 | 100149189 100000252\_3 30762 58113 368878 100330617\_1 768248 561021 30763 791704 100000252 571282 564266 556826\_1 | si:ch73-237c6.1 pik3r3b mhc2dab cd74a si:busm1-266f07.2 tsc1b fas si:dkey-103e21.5 mhc2dcb zgc:153759 pik3r3b LOC571282 itgb3a si:dkey-57h18.2 | dre:100149189 dre:100000252 dre:30762 dre:58113 dre:368878 dre:100330617 dre:768248 dre:561021 dre:30763 dre:791704 dre:100000252 dre:571282 dre:564266 dre:556826 |
| dre00650 | Butanoate metabolism | 2/511 | 26/8379 | 0.476668862585525 | 0.963031711752537 | 797311 565235\_1 | LOC797311 aldh5a1 | dre:797311 dre:565235 |
| dre04912 | GnRH signaling pathway | 10/511 | 158/8379 | 0.499061114788367 | 0.963031711752537 | 336121 30498\_1 562552 556410\_1 793158\_1 100006675 568726 556226\_1 565743\_1 560807 | calm2a egr1 zgc:171775 atf4b cdc42l prkcba adcy7 cdc42l2 pld2 adcy6b | dre:192322 dre:30498 dre:562552 dre:556410 dre:793158 dre:100006675 dre:568726 dre:556226 dre:565743 dre:560807 |
| dre00740 | Riboflavin metabolism | 1/511 | 11/8379 | 0.499725387195105 | 0.963031711752537 | 436725\_1 | acp5b | dre:436725 |
| dre00230 | Purine metabolism | 12/511 | 192/8379 | 0.508299039180196 | 0.963031711752537 | 100330864 334431\_1 560827\_1 568726 550560\_1 553639\_1 402953\_1 333997 560807 447905 140425 333997\_1 | LOC100330864 adsl prps1b adcy7 entpd6 gmpr pnp6 ampd3b adcy6b entpd2a.1 gc2 ampd3b | dre:100330864 dre:334431 dre:560827 dre:568726 dre:550560 dre:553639 dre:402953 dre:333997 dre:560807 dre:447905 dre:140425 dre:333997 |
| dre04145 | Phagosome | 13/511 | 209/8379 | 0.512350973991672 | 0.963031711752537 | 57934\_1 359840 100004793 30762 368878 100537272 403014 30763 431777 359839 571282 564266 556826\_1 | actb1 atp6v1b2 mrc2 mhc2dab si:busm1-266f07.2 LOC100537272 ncf4 mhc2dcb tuba7l atp6v1ba LOC571282 itgb3a si:dkey-57h18.2 | dre:57934 dre:359840 dre:100004793 dre:30762 dre:368878 dre:100537272 dre:403014 dre:30763 dre:431777 dre:359839 dre:571282 dre:564266 dre:556826 |
| dre04270 | Vascular smooth muscle contraction | 14/511 | 226/8379 | 0.51611243055152 | 0.963031711752537 | 337226\_1 336121 436718 798632 406370 100006675 436966\_2 568726 403114\_1 567131 393783\_1 560807 492486 101882911 | zgc:153867 calm2a rhocb myl6 pla2g6 prkcba zgc:91818 adcy7 adm2a calcrlb ppp1r14ab adcy6b adra1bb LOC101882911 | dre:337226 dre:192322 dre:436718 dre:798632 dre:406370 dre:100006675 dre:436966 dre:568726 dre:403114 dre:567131 dre:393783 dre:560807 dre:492486 dre:321070 |
| dre00770 | Pantothenate and CoA biosynthesis | 2/511 | 28/8379 | 0.516353731580427 | 0.963031711752537 | 368239 406789 | aldh2.2 pank1a | dre:368239 dre:406789 |
| dre04520 | Adherens junction | 12/511 | 194/8379 | 0.523092451191501 | 0.963031711752537 | 57934\_1 566841 436718 793158\_1 677750 335573\_1 556226\_1 393527 30429 245702 564805\_1 101882911 | actb1 crebbpa rhocb cdc42l myl2b ptpn6 cdc42l2 pdcd10a mylpfa igf1rb arhgap29a LOC101882911 | dre:57934 dre:566841 dre:436718 dre:793158 dre:677750 dre:335573 dre:556226 dre:393527 dre:30429 dre:245702 dre:378998 dre:321070 |
| dre04512 | ECM-receptor interaction | 8/511 | 128/8379 | 0.524147695511907 | 0.963031711752537 | 570868\_1 563428\_1 321243\_1 30037 798998 322012\_1 100005469\_1 564266 | itga1 fras1 lama5 tnc gp1bb hmmr fn1a itgb3a | dre:570868 dre:563428 dre:321243 dre:30037 dre:798998 dre:322012 dre:100005469 dre:564266 |
| dre00920 | Sulfur metabolism | 1/511 | 12/8379 | 0.530275139482786 | 0.963031711752537 | 393542\_1 | selenbp1 | dre:393542 |
| dre00062 | Fatty acid elongation | 3/511 | 46/8379 | 0.538363317912269 | 0.963031711752537 | 393441 554145 393657 | hacd3 elovl8b zgc:66024 | dre:393441 dre:554145 dre:393657 |
| dre00630 | Glyoxylate and dicarboxylate metabolism | 3/511 | 46/8379 | 0.538363317912269 | 0.963031711752537 | 335715 437019\_1 541329 | mdh1ab pcca gcsha | dre:335715 dre:437019 dre:541329 |
| dre04146 | Peroxisome | 7/511 | 114/8379 | 0.548626494275499 | 0.963031711752537 | 100093708\_1 100006589\_1 405800 406803\_1 406803\_2 100003383 619259 | slc25a17 idh1 dao.2 abcd3a abcd3a pmvk dao.1 | dre:100093708 dre:100006589 dre:405800 dre:406803 dre:406803 dre:100003383 dre:619259 |
| dre00340 | Histidine metabolism | 2/511 | 30/8379 | 0.553991058854808 | 0.963031711752537 | 368239 555401 | aldh2.2 aoc1 | dre:368239 dre:555401 |
| dre01210 | 2-Oxocarboxylic acid metabolism | 2/511 | 30/8379 | 0.553991058854808 | 0.963031711752537 | 100006589\_1 550579\_1 | idh1 idh3g | dre:100006589 dre:550579 |
| dre04120 | Ubiquitin mediated proteolysis | 12/511 | 199/8379 | 0.559455242557159 | 0.963031711752537 | 192341 550134\_1 692325 564149\_1 novel.905 323951\_2 556277 564866\_1 559439\_1 393445\_1 559981 100151173\_1 | tceb2 ubb flj11011l pias2 - klhl13 cblb trip12 huwe1 ube3c si:ch73-190m4.1 si:rp71-68n21.9 | dre:192341 dre:550134 dre:692325 dre:564149 dre:777766 dre:323951 dre:556277 dre:564866 dre:559439 dre:393445 dre:559981 dre:100151173 |
| dre00514 | Other types of O-glycan biosynthesis | 4/511 | 65/8379 | 0.566190372417317 | 0.963031711752537 | 556077\_1 557675\_1 555344 337131 | plod3 c1galt1a si:dkey-202e17.1 c1galt1b | dre:556077 dre:557675 dre:555344 dre:337131 |
| dre03050 | Proteasome | 4/511 | 65/8379 | 0.566190372417317 | 0.963031711752537 | 30387 796450\_1 326884\_1 436816 | psmb5 adrm1 psmc4 zgc:92791 | dre:30387 dre:796450 dre:326884 dre:436816 |
| dre03030 | DNA replication | 3/511 | 48/8379 | 0.567625103529318 | 0.963031711752537 | 393241\_1 393774 556995\_1 | rnaseh2b pole3 lig1 | dre:393241 dre:393774 dre:556995 |
| dre00532 | Glycosaminoglycan biosynthesis - chondroitin sulfate / dermatan sulfate | 2/511 | 31/8379 | 0.572017672276479 | 0.963031711752537 | 100331876 100538167 | LOC100331876 si:dkey-26i13.6 | dre:100331876 dre:100538167 |
| dre03008 | Ribosome biogenesis in eukaryotes | 5/511 | 83/8379 | 0.576985110486915 | 0.963031711752537 | 393107\_2 407084\_1 393107\_1 492761\_1 553787\_1 | utp6 riok2 utp6 tbl3 pop4 | dre:393107 dre:407084 dre:393107 dre:492761 dre:553787 |
| dre04510 | Focal adhesion | 21/511 | 354/8379 | 0.586330761511702 | 0.963031711752537 | 57934\_1 562529\_1 570868\_1 436718 321243\_1 563023\_1 793158\_1 100000252\_3 100006675 30037 677750 556226\_1 796490\_2 100005469\_1 30429 100000252 245702 103909601 564565 564266 101882911 | actb1 flna itga1 rhocb lama5 flnb cdc42l pik3r3b prkcba tnc myl2b cdc42l2 pdgfbb fn1a mylpfa pik3r3b igf1rb LOC103909601 arhgap35 itgb3a LOC101882911 | dre:57934 dre:562529 dre:570868 dre:436718 dre:321243 dre:563023 dre:793158 dre:100000252 dre:100006675 dre:30037 dre:677750 dre:556226 dre:796490 dre:100005469 dre:30429 dre:100000252 dre:245702 dre:103909601 dre:564565 dre:564266 dre:321070 |
| dre04115 | p53 signaling pathway | 6/511 | 101/8379 | 0.586390345557505 | 0.963031711752537 | 100330864 431763 171473 436699\_1 768248 561021 | LOC100330864 gadd45ab ccng1 zgc:92658 fas si:dkey-103e21.5 | dre:100330864 dre:431763 dre:171473 dre:436699 dre:768248 dre:561021 |
| dre04514 | Cell adhesion molecules | 12/511 | 204/8379 | 0.594731038180354 | 0.963031711752537 | 30194 30762 793143 30634\_1 368878 797651\_1 30763 571282 541474 100151365\_1 100005566 562934\_1 | alcama mhc2dab si:dkey-98f17.3 nadl1.2 si:busm1-266f07.2 jam3a mhc2dcb LOC571282 cntn1b si:dkey-1h24.6 f11r.2 esamb | dre:30194 dre:30762 dre:793143 dre:30634 dre:368878 dre:797651 dre:30763 dre:571282 dre:541474 dre:100151365 dre:100005566 dre:562934 |
| dre04916 | Melanogenesis | 10/511 | 171/8379 | 0.601521070689366 | 0.963031711752537 | 323509\_1 336121 566841 100006675 568726 560807 100538072 556087 553181\_1 58072\_1 | gnaia calm2a crebbpa prkcba adcy7 adcy6b creb3l3b wnt2bb kitlgb fzd8b | dre:323509 dre:192322 dre:566841 dre:100006675 dre:568726 dre:560807 dre:100538072 dre:556087 dre:553181 dre:58072 |
| dre01250 | Biosynthesis of nucleotide sugars | 3/511 | 51/8379 | 0.609314773576686 | 0.963031711752537 | 100885772 321224 751668 | cmasb hkdc1 gck | dre:100885772 dre:321224 dre:751668 |
| dre04140 | Autophagy - animal | 14/511 | 241/8379 | 0.615089719647183 | 0.963031711752537 | 110438871 792229\_1 449923 100000252\_3 326974\_1 567021 449923\_1 569298 100330617\_1 563140 406843\_1 100000252 245702 573492\_1 | LOC110438871 sh3glb1a si:ch73-234b20.5 pik3r3b gabarapa ern2 si:ch73-234b20.5 ctsbb tsc1b si:ch211-11n16.2 igbp1 pik3r3b igf1rb rragca | dre:110438871 dre:792229 dre:449923 dre:100000252 dre:326974 dre:567021 dre:449923 dre:569298 dre:100330617 dre:563140 dre:406843 dre:100000252 dre:245702 dre:573492 |
| dre00760 | Nicotinate and nicotinamide metabolism | 3/511 | 52/8379 | 0.622601003676548 | 0.963031711752537 | 402953\_1 101886891 110438097 | pnp6 LOC101886891 LOC110438097 | dre:402953 dre:101886891 dre:572070 |
| dre03450 | Non-homologous end-joining | 1/511 | 16/8379 | 0.634951888544244 | 0.963031711752537 | 566285\_1 | dclre1c | dre:566285 |
| dre00983 | Drug metabolism - other enzymes | 7/511 | 124/8379 | 0.638519835492272 | 0.963031711752537 | 100330864 492517 100005763 103910413\_1 393493 558466\_1 431762\_1 | LOC100330864 zgc:103586 uck2a tymp gstt2 uckl1b mgst1.2 | dre:100330864 dre:492517 dre:100005763 dre:103910413 dre:393493 dre:558466 dre:431762 |
| dre04623 | Cytosolic DNA-sensing pathway | 4/511 | 72/8379 | 0.647673846367237 | 0.963031711752537 | 562329 566185 394162\_1 561021 | polr3b caspbl polr3f si:dkey-103e21.5 | dre:562329 dre:566185 dre:394162 dre:561021 |
| dre01212 | Fatty acid metabolism | 5/511 | 91/8379 | 0.658971107946117 | 0.963031711752537 | 140615 393441 559403\_1 554145 393657 | fads2 hacd3 acaca elovl8b zgc:66024 | dre:140615 dre:393441 dre:559403 dre:554145 dre:393657 |
| dre04261 | Adrenergic signaling in cardiomyocytes | 17/511 | 301/8379 | 0.66581948338766 | 0.963031711752537 | 323509\_1 336121 562552 556410\_1 793666\_1 100535196 568726 555423\_1 677750 794522 559392\_1 562282 114457\_1 560807 492486 100538072 558525\_1 | gnaia calm2a zgc:171775 atf4b popdc2 cacna2d3a adcy7 scn1ba myl2b si:ch211-139a5.9 ppp2r2cb ppp2r5b atp1b2b adcy6b adra1bb creb3l3b atp2b3b | dre:323509 dre:192322 dre:562552 dre:556410 dre:793666 dre:100535196 dre:568726 dre:555423 dre:677750 dre:794522 dre:559392 dre:562282 dre:114457 dre:560807 dre:492486 dre:100538072 dre:558525 |
| dre04218 | Cellular senescence | 13/511 | 234/8379 | 0.677477537946725 | 0.963031711752537 | 333941\_1 336121 393186\_1 431763 562552 767751\_1 100000252\_3 393176 557551\_1 100330617\_1 565241 100000252 559723 | zfp36l2 calm2a zgc:56235 gadd45ab zgc:171775 ccnb3 pik3r3b rad9a hipk2 tsc1b ppp3ccb pik3r3b tgfb2l | dre:333941 dre:192322 dre:393186 dre:431763 dre:562552 dre:767751 dre:100000252 dre:393176 dre:557551 dre:100330617 dre:565241 dre:100000252 dre:559723 |
| dre04114 | Oocyte meiosis | 10/511 | 182/8379 | 0.679697017482842 | 0.963031711752537 | 336121 562552 568726 562282 565241 560807 245702 100009657\_1 100005148\_1 280649\_1 | calm2a zgc:171775 adcy7 ppp2r5b ppp3ccb adcy6b igf1rb rps6ka3b ar plk1 | dre:192322 dre:562552 dre:568726 dre:562282 dre:565241 dre:560807 dre:245702 dre:100009657 dre:100005148 dre:280649 |
| dre00534 | Glycosaminoglycan biosynthesis - heparan sulfate / heparin | 2/511 | 38/8379 | 0.68326091730005 | 0.963031711752537 | 792524 791107\_1 | hs6st3a hs6st1b | dre:792524 dre:791107 |
| dre00250 | Alanine, aspartate and glutamate metabolism | 3/511 | 57/8379 | 0.684325224937276 | 0.963031711752537 | 334431\_1 447815 565235\_1 | adsl rimkla aldh5a1 | dre:334431 dre:447815 dre:565235 |
| dre04744 | Phototransduction | 3/511 | 57/8379 | 0.684325224937276 | 0.963031711752537 | 336121 792319 140425 | calm2a saga gc2 | dre:192322 dre:792319 dre:140425 |
| dre04622 | RIG-I-like receptor signaling pathway | 4/511 | 76/8379 | 0.689427841484501 | 0.963031711752537 | 562552 561021 393144\_2 100148871 | zgc:171775 si:dkey-103e21.5 trim25 dhx58 | dre:562552 dre:561021 dre:393144 dre:100148871 |
| dre03410 | Base excision repair | 3/511 | 58/8379 | 0.695723772399993 | 0.963031711752537 | 110438871 393774 556995\_1 | LOC110438871 pole3 lig1 | dre:110438871 dre:393774 dre:556995 |
| dre00604 | Glycosphingolipid biosynthesis - ganglio series | 1/511 | 19/8379 | 0.697869515172089 | 0.963031711752537 | 394230 | st3gal5 | dre:394230 |
| dre00410 | beta-Alanine metabolism | 2/511 | 41/8379 | 0.723169511976643 | 0.963031711752537 | 541429 368239 | aoc2 aldh2.2 | dre:541429 dre:368239 |
| dre04136 | Autophagy - other | 2/511 | 41/8379 | 0.723169511976643 | 0.963031711752537 | 326974\_1 406843\_1 | gabarapa igbp1 | dre:326974 dre:406843 |
| dre04371 | Apelin signaling pathway | 12/511 | 226/8379 | 0.732314353137284 | 0.963031711752537 | 323509\_1 117508\_1 336121 30498\_1 117508 321449 100331581 568726 677750 560807 557486\_1 335655\_1 | gnaia klf2a calm2a egr1 klf2a ctgfa si:dkey-44g17.6 adcy7 myl2b adcy6b hdac5 gngt2a | dre:323509 dre:117508 dre:192322 dre:30498 dre:117508 dre:321449 dre:100331581 dre:568726 dre:677750 dre:560807 dre:557486 dre:335655 |
| dre03020 | RNA polymerase | 2/511 | 42/8379 | 0.735496337851617 | 0.963031711752537 | 562329 394162\_1 | polr3b polr3f | dre:562329 dre:394162 |
| dre04530 | Tight junction | 16/511 | 300/8379 | 0.748257234504753 | 0.963031711752537 | 57934\_1 337226\_1 286739 436718 798632 793158\_1 792828\_1 677750 556226\_1 793143 797651\_1 559392\_1 431777 793952\_1 100005566 101882911 | actb1 zgc:153867 msna rhocb myl6 cdc42l pard6a myl2b cdc42l2 si:dkey-98f17.3 jam3a ppp2r2cb tuba7l si:dkey-253i9.4 f11r.2 LOC101882911 | dre:57934 dre:337226 dre:286739 dre:436718 dre:798632 dre:793158 dre:792828 dre:677750 dre:556226 dre:793143 dre:797651 dre:559392 dre:431777 dre:793952 dre:100005566 dre:321070 |
| dre04110 | Cell cycle | 12/511 | 229/8379 | 0.748488254657022 | 0.963031711752537 | 560751\_1 431763 566841 327226 767751\_1 554509\_1 100141368\_1 794108\_1 562282 327060 280649\_1 559723 | stag2b gadd45ab crebbpa casc5 ccnb3 orc1 waplb nipblb ppp2r5b wee1 plk1 tgfb2l | dre:560751 dre:431763 dre:566841 dre:327226 dre:767751 dre:554509 dre:100141368 dre:794108 dre:562282 dre:327060 dre:280649 dre:559723 |
| dre04620 | Toll-like receptor signaling pathway | 6/511 | 121/8379 | 0.755553149296904 | 0.963031711752537 | 562552 100000252\_3 394198 570238 561021 100000252 | zgc:171775 pik3r3b fosab fosaa si:dkey-103e21.5 pik3r3b | dre:562552 dre:100000252 dre:394198 dre:570238 dre:561021 dre:100000252 |
| dre00430 | Taurine and hypotaurine metabolism | 1/511 | 23/8379 | 0.765249537912961 | 0.963031711752537 | 794311 | fmo5 | dre:794311 |
| dre00071 | Fatty acid degradation | 3/511 | 65/8379 | 0.766879602918535 | 0.963031711752537 | 116517 368239 393860 | adh5 aldh2.2 gcdha | dre:116517 dre:368239 dre:393860 |
| dre04540 | Gap junction | 8/511 | 160/8379 | 0.768090082497139 | 0.963031711752537 | 323509\_1 100006675 568726 796490\_2 431777 560807 103909601 559806 | gnaia prkcba adcy7 pdgfbb tuba7l adcy6b LOC103909601 htr2ab | dre:323509 dre:100006675 dre:568726 dre:796490 dre:431777 dre:560807 dre:103909601 dre:559806 |
| dre00830 | Retinol metabolism | 5/511 | 104/8379 | 0.768618912177346 | 0.963031711752537 | 116517 562840 553969 445083 393580 | adh5 rdh10a cyp3a65 dhrs3a bco1l | dre:116517 dre:562840 dre:553969 dre:445083 dre:393580 |
| dre03022 | Basal transcription factors | 2/511 | 45/8379 | 0.769700974555479 | 0.963031711752537 | 798997\_1 798997 | gtf2a2 gtf2a2 | dre:798997 dre:798997 |
| dre00982 | Drug metabolism - cytochrome P450 | 4/511 | 85/8379 | 0.77017016590788 | 0.963031711752537 | 116517 393493 431762\_1 794311 | adh5 gstt2 mgst1.2 fmo5 | dre:116517 dre:393493 dre:431762 dre:794311 |
| dre00053 | Ascorbate and aldarate metabolism | 3/511 | 66/8379 | 0.775852306065794 | 0.963031711752537 | 403070\_1 368239 445326\_1 | rgn aldh2.2 akr1a1a | dre:403070 dre:368239 dre:445326 |
| dre03420 | Nucleotide excision repair | 3/511 | 68/8379 | 0.792947625633006 | 0.963031711752537 | 393774 556995\_1 100006257 | pole3 lig1 cetn2 | dre:393774 dre:556995 dre:100006257 |
| dre00563 | Glycosylphosphatidylinositol (GPI)-anchor biosynthesis | 1/511 | 25/8379 | 0.793084939090325 | 0.963031711752537 | 561471\_1 | pigl | dre:561471 |
| dre00670 | One carbon pool by folate | 1/511 | 25/8379 | 0.793084939090325 | 0.963031711752537 | 557552 | zgc:153031 | dre:557552 |
| dre04621 | NOD-like receptor signaling pathway | 10/511 | 205/8379 | 0.810407598384878 | 0.963177591291292 | 393186\_1 562552 436718 326974\_1 569298 566185 557239\_1 561021 110437957 110438502 | zgc:56235 zgc:171775 rhocb gabarapa ctsbb caspbl antxr2a si:dkey-103e21.5 LOC110437957 LOC110438502 | dre:393186 dre:562552 dre:436718 dre:326974 dre:569298 dre:566185 dre:557239 dre:561021 dre:373874 dre:796505 |
| dre00531 | Glycosaminoglycan degradation | 1/511 | 27/8379 | 0.817625462375716 | 0.963177591291292 | 567720 | idua | dre:567720 |
| dre00970 | Aminoacyl-tRNA biosynthesis | 2/511 | 51/8379 | 0.826697335996423 | 0.963177591291292 | 101884223 792787 | LOC101884223 aars2 | dre:101884223 dre:792787 |
| dre00061 | Fatty acid biosynthesis | 1/511 | 28/8379 | 0.828783674627393 | 0.963177591291292 | 559403\_1 | acaca | dre:559403 |
| dre00511 | Other glycan degradation | 1/511 | 28/8379 | 0.828783674627393 | 0.963177591291292 | 566517 | aga | dre:566517 |
| dre04216 | Ferroptosis | 3/511 | 73/8379 | 0.830976745427781 | 0.963177591291292 | 796322 393186\_1 450084\_1 | slc3a2a zgc:56235 gss | dre:796322 dre:393186 dre:450084 |
| dre00520 | Amino sugar and nucleotide sugar metabolism | 3/511 | 74/8379 | 0.837820762328189 | 0.96380884688882 | 100885772 321224 751668 | cmasb hkdc1 gck | dre:100885772 dre:321224 dre:751668 |
| dre04217 | Necroptosis | 10/511 | 215/8379 | 0.852988305049372 | 0.972777754579027 | 110438871 393444 336426 393186\_1 337413\_1 566185 768248 561021 100149436 100148591 | LOC110438871 pygmb chmp1b zgc:56235 stat6 caspbl fas si:dkey-103e21.5 si:ch211-189a15.5 LOC100148591 | dre:110438871 dre:393444 dre:336426 dre:393186 dre:337413 dre:566185 dre:768248 dre:561021 dre:100149436 dre:561187 |
| dre00120 | Primary bile acid biosynthesis | 1/511 | 31/8379 | 0.858333312863847 | 0.972777754579027 | 641477 | zgc:123299 | dre:553543 |
| dre04141 | Protein processing in endoplasmic reticulum | 11/511 | 241/8379 | 0.877504894191207 | 0.981164779190726 | 386590\_1 327197\_1 266757 573376 337190 567029\_1 566876 556410\_1 567021 108181182 574002 | hsp90b1 vcp tram1 hspa8 ssr3 rrbp1b wfs1a atf4b ern2 LOC108181182 zgc:114174 | dre:386590 dre:327197 dre:266757 dre:573376 dre:337190 dre:567029 dre:566876 dre:556410 dre:567021 dre:567029 dre:574002 |
| dre04020 | Calcium signaling pathway | 22/511 | 450/8379 | 0.888394364061916 | 0.981164779190726 | 336121 393186\_1 368717 544664\_1 100006675 568726 796490\_2 494456 58147 101886891 565241 58133 492486 794365 100005590 103909601 445226 558525\_1 555941 559806 569706\_1 100137117\_1 | calm2a zgc:56235 itpka erbb3b prkcba adcy7 pdgfbb fgf23 p2rx3a LOC101886891 ppp3ccb ngfb adra1bb si:dkey-163m14.7 hrh2b LOC103909601 casq1a atp2b3b cacna1ba htr2ab phkb si:dkey-1h24.2 | dre:192322 dre:393186 dre:368717 dre:100149258 dre:100006675 dre:568726 dre:796490 dre:494456 dre:58147 dre:101886891 dre:565241 dre:58133 dre:492486 dre:794365 dre:100005590 dre:103909601 dre:445226 dre:558525 dre:555941 dre:559806 dre:569706 dre:100137117 |
| dre04012 | ErbB signaling pathway | 5/511 | 127/8379 | 0.89413547085157 | 0.981164779190726 | 544664\_1 100000252\_3 100006675 556277 100000252 | erbb3b pik3r3b prkcba cblb pik3r3b | dre:100149258 dre:100000252 dre:100006675 dre:556277 dre:100000252 |
| dre00980 | Metabolism of xenobiotics by cytochrome P450 | 3/511 | 85/8379 | 0.898577575811612 | 0.981164779190726 | 116517 393493 431762\_1 | adh5 gstt2 mgst1.2 | dre:116517 dre:393493 dre:431762 |
| dre00040 | Pentose and glucuronate interconversions | 2/511 | 62/8379 | 0.899244683442802 | 0.981164779190726 | 445326\_1 393350 | akr1a1a xylb | dre:445326 dre:393350 |
| dre00190 | Oxidative phosphorylation | 7/511 | 171/8379 | 0.904210678862042 | 0.981164779190726 | 447914\_1 573619 359840 767750\_2 447942 767750 359839 | cox17 ndufab1a atp6v1b2 atp5ib cox6a2 atp5ib atp6v1ba | dre:447914 dre:573619 dre:359840 dre:767750 dre:447942 dre:767750 dre:359839 |
| dre03010 | Ribosome | 6/511 | 161/8379 | 0.933694063117768 | 0.984402592455853 | 336712\_2 415196\_1 327089 553284\_1 560828\_1 337007\_1 | rpl10 rpl5b rpl12 mrpl1 rpl13a rps12 | dre:336712 dre:415196 dre:327089 dre:553284 dre:560828 dre:337007 |
| dre04814 | Motor proteins | 13/511 | 305/8379 | 0.937830138643917 | 0.984402592455853 | 57934\_1 337226\_1 798632 101882034 474327 677750 556023\_1 799461\_2 431777 30429 571487 562052\_1 100334351 | actb1 zgc:153867 myl6 LOC101882034 capza1b myl2b dnah2 dnah12 tuba7l mylpfa si:dkeyp-92c9.3 kif14 dnah3 | dre:57934 dre:337226 dre:798632 dre:406297 dre:474327 dre:677750 dre:556023 dre:799461 dre:431777 dre:30429 dre:571487 dre:562052 dre:100334351 |
| dre03040 | Spliceosome | 6/511 | 164/8379 | 0.940533217683821 | 0.984402592455853 | 100004402\_1 100037380\_3 573376 100005893 566017\_1 334283\_2 | plrg1 zgc:163098 hspa8 magoh lsm8 ddx23 | dre:100004402 dre:100037380 dre:573376 dre:100005893 dre:566017 dre:334283 |
| dre00860 | Porphyrin metabolism | 2/511 | 74/8379 | 0.945571449491576 | 0.984402592455853 | 100002875 101886332 | hmox2a hephl1b | dre:100002875 dre:101886332 |
| dre04310 | Wnt signaling pathway | 11/511 | 278/8379 | 0.957324350970183 | 0.984402592455853 | 678649\_1 566841 436718 100000500\_1 100006675 564241 103909653 565241 556087 58072\_1 101882911 | zgc:136929 crebbpa rhocb sost prkcba fosl1a LOC103909653 ppp3ccb wnt2bb fzd8b LOC101882911 | dre:678649 dre:566841 dre:436718 dre:100000500 dre:100006675 dre:564241 dre:103909653 dre:565241 dre:556087 dre:58072 dre:321070 |
| dre04260 | Cardiac muscle contraction | 6/511 | 173/8379 | 0.957435039824359 | 0.984402592455853 | 100535196 447942 677750 556638\_2 794522 114457\_1 | cacna2d3a cox6a2 myl2b slc9a6b si:ch211-139a5.9 atp1b2b | dre:100535196 dre:447942 dre:677750 dre:556638 dre:794522 dre:114457 |
| dre03250 | Viral life cycle - HIV-1 | 2/511 | 83/8379 | 0.966143943317989 | 0.984402592455853 | 566841 64275\_1 | crebbpa supt5h | dre:566841 dre:64275 |
| dre04350 | TGF-beta signaling pathway | 5/511 | 161/8379 | 0.97191552036808 | 0.984402592455853 | 566841 436718 100004116\_1 405778 559723 | crebbpa rhocb fsta grem1a tgfb2l | dre:566841 dre:436718 dre:100004116 dre:100332300 dre:559723 |
| dre04144 | Endocytosis | 17/511 | 417/8379 | 0.975013117442333 | 0.984402592455853 | 792229\_1 573376 336426 406837\_1 436718 793158\_1 336368 406684\_1 792828\_1 393881 474327 556226\_1 556277 565743\_1 568170\_1 561771 245702 | sh3glb1a hspa8 chmp1b rnf41 rhocb cdc42l vps37a vps26b pard6a vps26a capza1b cdc42l2 cblb pld2 eps15l1a acap1 igf1rb | dre:792229 dre:573376 dre:336426 dre:406837 dre:436718 dre:793158 dre:336368 dre:406684 dre:792828 dre:393881 dre:474327 dre:556226 dre:556277 dre:565743 dre:568170 dre:561771 dre:245702 |
| dre00600 | Sphingolipid metabolism | 2/511 | 89/8379 | 0.975455352902841 | 0.984402592455853 | 450083 493602 | gla asah2 | dre:450083 dre:493602 |
| dre00140 | Steroid hormone biosynthesis | 2/511 | 91/8379 | 0.977968588583593 | 0.984402592455853 | 553969 791578\_1 | cyp3a65 dhrs11a | dre:553969 dre:791578 |
| dre04920 | Adipocytokine signaling pathway | 2/511 | 108/8379 | 0.991329567988305 | 0.991329567988305 | 406473 100149042 | pck2 agrp | dre:406473 dre:100149042 |
